# Deciphering “False Maturity” in Mountain Coffee: A Multimodal Hyperspectral Framework for Non-Destructive Sugar Content Assessment

**DOI:** 10.3390/foods15122149

**Published:** 2026-06-14

**Authors:** Hongbo Zhao, Zhijia Wang, Linrui Deng, Huijuan Yang, Luoyi Zheng, Guangyao Jian, Jiyuan Cai, Yuanhao Zhang, Zhiyong Cao

**Affiliations:** College of Big Data, Yunnan Agricultural University, Kunming 650201, China2024240729@stu.ynau.edu.cn (L.D.); 18388254083@163.com (G.J.); tommy_nike@163.com (J.C.); 15391434766@163.com (Y.Z.)

**Keywords:** mountain coffee, hyperspectral imaging, multimodal fusion, false maturity, postharvest sorting

## Abstract

In complex mountainous environments, the asynchronous development between external color turning and internal sugar accumulation (often termed “false maturity”) in coffee cherries poses a severe challenge to post-harvest quality sorting and the consistency of final coffee products. To overcome the limitations of single-phenotype detection in raw material screening, this study proposed a multimodal quality discrimination framework integrating fruit hyperspectral imaging, micro-topography, and plant physiological characteristics. Taking typical mountain-grown fresh coffee cherries as the research object, and after comparing various spectral preprocessing and feature dimensionality reduction algorithms, the multimodal fusion efficacy of nine machine learning classifiers was systematically evaluated. The results demonstrated that: (1) Full-spectrum difference analysis quantitatively confirmed the limitations of visual harvesting; spectral reflectance differences between high- and low-sugar fruits were highly concentrated in the red and red-edge regions, with the maximum difference precisely located at 676 nm. (2) Compared to the single-spectrum model (mean accuracy of 75.93%), the fully fused Multilayer Perceptron (MLP) network effectively mitigated background noise induced by heterogeneous environments, improving the mean classification accuracy to 77.22% with a mean Area Under the Curve (AUC) of 0.827. (3) Correlation analysis clarified the quantitative association between topography and quality; micro-topographic slope (r = 0.346) was identified as the key environmental driver of spatial differentiation in fruit sugar content, while plant chlorophyll A content (r = 0.183) exhibited a corresponding physiological response trend. This study not only explains the root cause of visual assessment failure from a physical optics perspective but also reveals the spatial variation laws of quality driven by micro-topography, providing preliminary data support for the intelligent sorting of raw materials and ensuring post-harvest quality consistency of mountainous crops.

## 1. Introduction

Coffee is one of the most traded tropical cash crops globally and ranks among the most popular and frequently consumed beverages worldwide [1,2]. The ultimate flavor quality of coffee beans heavily relies on the dynamic changes of flavor precursors (e.g., sugars, amino acids) during the ripening process of coffee cherries [3]. Therefore, ensuring that the harvested cherries reach optimal maturity is the primary prerequisite for guaranteeing the production of specialty coffee.

In traditional production, farmers determine the optimal harvesting time by visually assessing the deepening of the red color on the cherry skin [4]. However, in mountainous coffee-growing regions, complex topography induces massive variations in microenvironments, such as light interception, temperature, and soil moisture [5]. These differentiated environmental stresses lead to an asynchronous progression between external skin coloration (secondary metabolism) and internal metabolic accumulation (primary metabolism), a phenomenon widely recognized in academia as “false maturity.” This asynchronous ripening renders color-based sorting strategies highly susceptible to the incorporation of low-quality raw materials that fail to meet standard sugar content levels. If these “false mature” cherries enter the subsequent post-harvest processing stages (e.g., fermentation, drying, and roasting) without being accurately screened out, the lack of sufficient flavor precursors (such as specific reducing sugars and amino acids) will severely restrict the final cup quality and economic profitability of mountain coffee [3]. Traditionally, the floating method is utilized to separate defective coffee cherries based on bulk density [6]. However, ‘false mature’ cherries are physically plump and share virtually identical density with truly mature cherries, rendering physical floating methods ineffective. Consequently, developing a method capable of penetrating the surface appearance to non-destructively evaluate the internal sugar content is crucial for screening out inferior raw materials at the source and implementing robust post-harvest quality control.

To achieve this goal, non-destructive sensing technologies are regarded as a paramount solution [7,8]. Furthermore, while conventional RGB and emerging ultraviolet (UV) imaging technologies are increasingly utilized, they are primarily sensitive to surface-level exocarp pigmentation [9]. Because false maturity involves asynchronous external color and internal synthesis, these surface sensors remain blind to internal quality. In contrast, hyperspectral imaging (HSI) in the near-infrared (NIR) region possesses distinct tissue penetrability, capturing the characteristic overtones of chemical bonds (such as O-H and C-H) inherent to soluble sugars, providing a fundamental molecular-level edge. Visible/Near-Infrared (Vis/NIR) spectroscopy, as a mature non-destructive detection tool, has been extensively utilized in the quality analysis of agricultural products, covering tasks such as peanut acidity detection, camellia seed oil content prediction, and flue-cured tobacco grading [10,11,12]. Notably, this technology has also been successfully applied to the variety classification and quality discrimination of green coffee beans [13], providing robust technological prior knowledge for its further application in the non-destructive assessment of the internal quality of fresh coffee cherries. Hyperspectral Imaging (HSI) integrates the advantages of both spectroscopic analysis and machine vision [14], enabling the simultaneous acquisition of spectral fingerprints and spatial distribution information of samples [15,16]. HSI has demonstrated tremendous potential in the quality detection of crops such as apples, tomatoes, and navel oranges [17,18,19].

However, most existing HSI-based non-destructive testing studies primarily focus on the precise continuous numerical prediction (regression analysis) of various physicochemical indices [17,19,20]. When confronting the complex and highly variable field environments of mountainous areas, relying solely on the spectral signals of fruits is extremely susceptible to background noise interference from abiotic environments, leading to severely restricted accuracy in quantitative prediction models. From the perspective of practical post-harvest quality control and industrial sorting, grading (classification) based on specific quality thresholds often possesses stronger anti-interference capability and greater engineering utility than precise numerical prediction. Furthermore, prevailing research frequently treats the fruit as an isolated detection object, overlooking the nonlinear interferences imposed by microhabitats on spectral features [21]. When traditional models extract only the fruit’s inherent signal while stripping away crucial background contextual information—such as plant physiology and growth environments that dictate the formation of these signals—their discriminative performance in complex environments deteriorates significantly [22]. In the context of intelligent sorting, utilizing these micro-environmental and physiological indicators as prior knowledge can effectively calibrate the spectral noise induced by heterogeneous field origins.

Based on the above premises, this study aimed to construct an analytical framework integrating multimodal information—plant physiology, micro-topography, and fruit hyperspectral data—and to explore an evaluation strategy shifting from precise numerical regression to comprehensive multimodal classification. This approach was designed to enhance the robustness and interpretability of hyperspectral technology in assessing mountain coffee quality. The specific objectives of this research were: (1) to quantitatively evaluate the indicating capability of conventional color features for internal sugar content under mountainous environments and verify the existence of the false maturity phenomenon at the hyperspectral level; (2) to compare the applicability of regression and classification models in complex environments and validate the effectiveness of incorporating environmental and physiological background information in improving classification performance; and (3) to dissect the core environmental factors driving the spatial variations in coffee quality and the false maturity phenomenon.

## 2. Materials and Methods

### 2.1. Data Collection Site and Equipment Description

**Figure 1:**
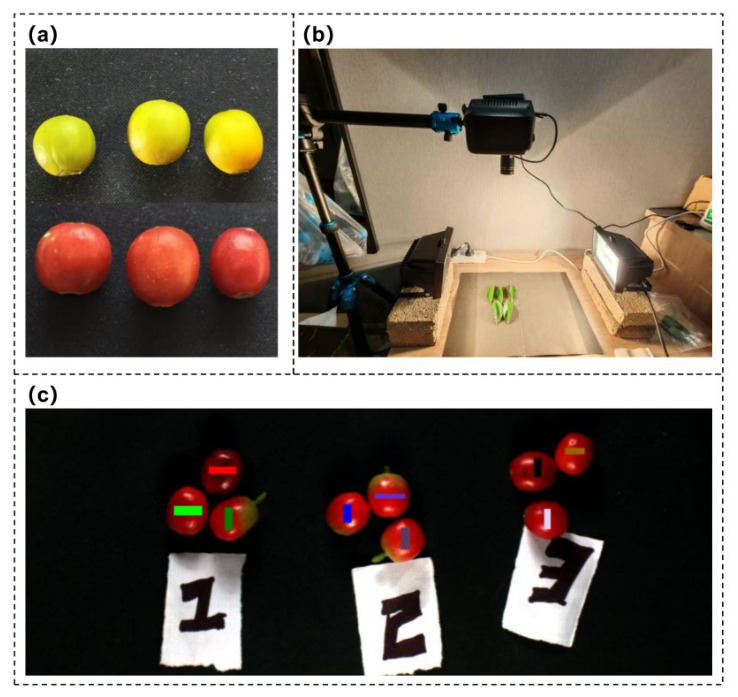
(**a**) Comparison of fruit appearance at different developmental stages; (**b**) Schematic diagram of the indoor hyperspectral image acquisition system; (**c**) Visualization of Region of Interest (ROI) extraction.

The field experiment was conducted during the 2025 growing season in a typical mountainous coffee plantation area located in Lujiangba, Baoshan City, Yunnan Province, China (98°44′–99°05′ E, 24°46′–25°33′ N), situated at an elevation of 1710 m above sea level. The research subjects were 10-year-old, healthy Typica (*Coffea arabica* var. Typica) coffee trees under uniform agricultural management. According to plantation records and field observations confirmed by the farm manager, all sampled trees originated from a single, locally maintained clonal lineage (propagated vegetatively), as evidenced by highly consistent phenotypic traits including low fruit load, uniform growth performance, and high susceptibility to pests and diseases. This clone represents a typical, yet genetically narrow, Typica ecotype in the Lujiangba region. This variety serves as a typical representative of Arabica (*Coffea arabica*) coffee [23]. Sampling spanned two critical phenological stages of fruit ripening: the color turning initiation stage (T1, the initial phase of chlorophyll degradation and sugar accumulation) and the fully mature stage (T2, where fruits are completely red and sugar content tends to stabilize) (Figure 1a). To capture the sample variance induced by complex mountainous microenvironments, a stratified random sampling strategy based on topographical factors was employed. Quadrats were delineated according to aspect and slope, and a total of 36 standard trees were selected. During each sampling period, three healthy cherries and three functional leaves were collected from the upper, middle, and lower canopy layers of each sampled tree, respectively, giving 9 cherries per tree. Cherries from the same layer of the same tree were pooled into one sample unit (3 cherries per unit), as single cherries are too small to yield sufficient juice volume for refractometer measurements. Thus, each tree contributed 3 sample units (one per layer). For the fully mature stage (T2), this resulted in 108 sample units (36 trees × 3 layers). Sample units from different trees were considered independent; units from different layers of the same tree were also treated as independent, based on the assumption that within-tree layer variation is comparable to among-tree variation. Potential non-independence (tree-level random effects) is acknowledged in Section 4.4. Ultimately, a total of 216 sample unit observations (T1 + T2) were obtained.

Hyperspectral image acquisition was performed using a portable hyperspectral camera (FS-IQ-VISNIR, spectral range: 400–1000 nm; CHNSpec, Zhejiang, China). The acquisition process was carried out in a controlled laboratory darkroom to simulate the standardized optical environment of industrial post-harvest sorting lines [24]. The system was equipped with two 150 W halogen lamps(OSRAM GmbH, Munich, Germany) positioned symmetrically at a 45° angle to illuminate the sample stage, ensuring uniform light distribution (Figure 1b).

### 2.2. Acquisition of Physicochemical Indices and Environmental Factors

#### 2.2.1. Determination of Fruit Sugar Content

The three coffee cherries collected from the same canopy layer during the T2 stage were pooled. Following standard field phenotyping protocols for fresh coffee, the seeds (beans) were manually extruded and excluded. The remaining peel, pulp, and mucilage were completely crushed, homogenized, and filtered. Their soluble solid content was then measured using a digital refractometer(DLX‑SDJ1514, DELIXI ELECTRIC, Zhejiang, China; 0–55% Brix), serving as the reference value for sugar content (°Brix). This mucilage-based measurement is a widely accepted agronomic proxy for internal sugar status, as the abundant sugars in the mucilage serve as critical substrates during post-harvest fermentation and fuel subsequent Maillard reactions during roasting.

#### 2.2.2. Measurement of Leaf Physiological Indices

Simultaneously with fruit harvesting, a portable plant nutrition meter (TYS-4N; Top Instrument, Hangzhou, China) was used in situ to measure the chlorophyll A, chlorophyll B, and nitrogen contents of the pooled leaves.

#### 2.2.3. Collection of Micro-Topographic Environmental Data

The geographical coordinates, aspect, and slope information for each sampled tree were recorded using a smartphone(Huawei Technologies Co., Ltd., Shenzhen, China) with built‑in GPS and digital compass.

### 2.3. Hyperspectral Data Extraction and Preprocessing

To calibrate for camera dark current and light source fluctuations, a standard black-and-white reference procedure was executed before and after each acquisition session. The dark current image (acquired with the lens covered), a high-reflectance (>99%) PTFE white reference image, and the sample images were sequentially collected. The reflectance was calculated according to Equation (1):

(1) $$R=\frac{R_{\mathrm{raw}\;}{-\;R}_{\mathrm{dark}}}{R_{\mathrm{white}\;}-\;R_{\mathrm{dark}}}$$

where $R_{\mathrm{raw}}$, $R_{\mathrm{dark}}$, and $R_{\mathrm{white}}$ represent the original, black reference, and white reference images, respectively. After calibration, rectangular Regions of Interest (ROIs) were manually delineated at the equatorial region of each cherry using ENVI 5.6 software (Figure 1c). The average reflectance of all pixels within the ROI was extracted as the representative spectrum of the fruit. All data processing and modeling used Python 3.12.10 (NumPy 1.26.0, Pandas 2.1.0, Matplotlib 3.8.0, SciPy 1.11.0, scikit‑learn 1.3.0, XGBoost 1.7.0, LightGBM 3.3.5). MATLAB R2025a was used for figure generation. ENVI 5.6 was used for ROI extraction.

Given that raw hyperspectral data are frequently contaminated with system noise, baseline drift, and light scattering interference [25], this study introduced multiple mathematical preprocessing algorithms for comparative evaluation. The evaluated algorithms covered nine mainstream methods, including Standard Normal Variate (SNV), Multiplicative Scatter Correction (MSC), First Derivative (1st-Der), and Second Derivative (2nd-Der) [26,27]. Among these, SNV and MSC are primarily utilized to eliminate the effects of light scattering caused by uneven particle distribution, whereas derivative algorithms effectively correct baseline drift and amplify overlapping spectral absorption peaks. This study aimed to identify the optimal preprocessing strategy for eliminating environmental optical noise based on the cross-validation performance of subsequent models.

### 2.4. Feature Selection Algorithms

Hyperspectral data are inherently characterized by a massive number of wavebands and strong multicollinearity among adjacent bands. To reduce the computational complexity of the models and eliminate redundant background information [28], this study introduced three distinct feature dimensionality reduction algorithms for performance comparison, based on the optimally preprocessed spectra. The CARS algorithm simulates the adaptive optimization mechanism of Darwinian evolution theory to select the most effective feature combinations within the spectrum [29]. The core concept of the UVE algorithm is to introduce random noise variables as a reference to identify and eliminate uninformative variables from the raw spectra [30]. SPA is a forward cyclical selection algorithm primarily aimed at minimizing multicollinearity in the vector space [31]. By comparing the effectiveness of these algorithms in retaining crucial pigment absorption peaks and chemical bond vibration information, the core set of characteristic wavelengths for subsequent modeling was ultimately established.

### 2.5. Source-Environment-Sink Multimodal Feature Fusion Framework

**Figure 2:**
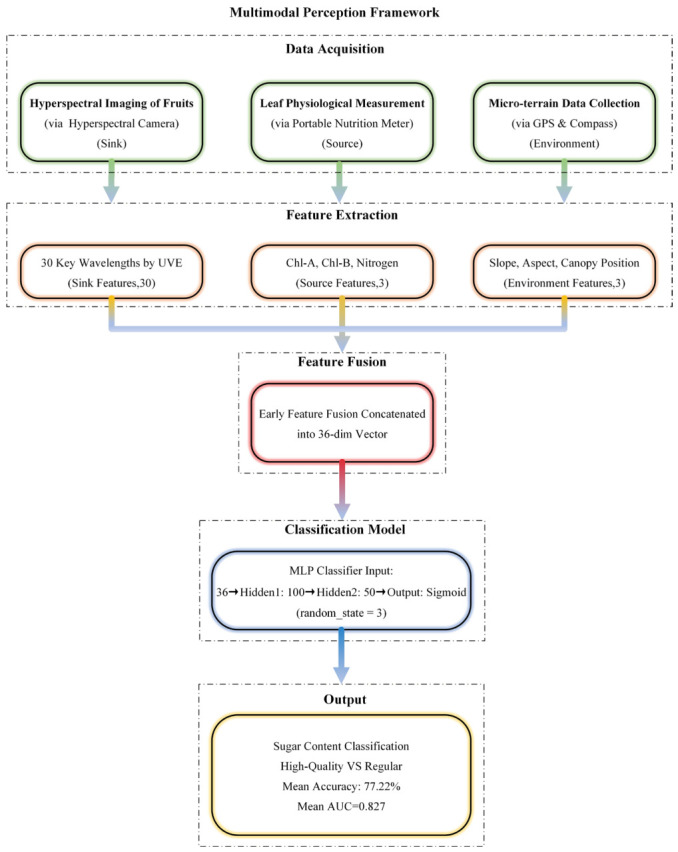
Multimodal perception framework.

Based on crop source-sink theory, this study constructed a data processing framework that integrates multi-source information (Figure 2). It specifically comprises features from three dimensions:

First, the internal fruit features (Sink): Namely, the dimensionality-reduced hyperspectral characteristic wavelengths, representing the internal metabolic state of the fruit.

Second, the growth environment features (Environment): Including slope, aspect, and canopy position, characterizing the microenvironmental stress variances during fruit development.

Third, the plant physiological features (Source): Including leaf chlorophyll A, chlorophyll B, and nitrogen contents, indicating the nutrient supply capacity of the plant.

These three distinct dimensions of data underwent early fusion at the feature level, concatenating into a high-dimensional comprehensive feature vector that served as the unified input for subsequent nonlinear classification models.

### 2.6. Model Construction and Evaluation

Given the inherent limitations of solely predicting absolute fruit sugar values in complex mountainous environments, this study divided the evaluation strategy into two stages: regression prediction and threshold-based classification. In the classification task, strictly referring to Arabica coffee sugar content benchmarks [32], 18.0 Brix was designated as the classification threshold for high-quality fruits.

For the core modeling task, 108 samples from the fully mature stage were extracted as the modeling dataset. Based on the established threshold, this dataset was objectively partitioned into 40 high-quality samples and 68 regular samples. Furthermore, rather than adopting the traditional fixed-ratio splitting strategy for training and testing sets, this study strictly employed a 10-fold stratified cross-validation mechanism.

In the regression prediction phase, nine algorithms were evaluated: PLSR, Random Forest, XGBoost, LightGBM, SVM, Gradient Boosting, MLP, Ridge, and Lasso. In the classification task phase, nine classifiers were compared: Random Forest, XGBoost, LightGBM, SVM, Logistic Regression, K-NN, Decision Tree, MLP, and Gradient Boosting. Prior to model training, cross-modal heterogeneous features were standardized to zero mean and unit variance via StandardScaler. StandardScaler was fitted on the training fold only and then applied to the corresponding validation fold; the same fitted scaler was used for the test fold within each cross-validation iteration.

To address the class imbalance, a dual strategy was adopted. For models that natively support cost-sensitive learning (e.g., Random Forest, SVM, Logistic Regression, Decision Tree), the parameter class_weight = ‘balanced’ was applied to heavily penalize misclassifications of the minority class. For the MLP classifier, which does not inherently accept class weights, we relied on stratified 10-fold cross-validation to preserve the original class distribution across all folds; additional synthetic weighting was deemed unnecessary given the relatively mild imbalance ratio (1:1.7). Furthermore, to ensure fairness in model evaluation, hyperparameters for the MLP classifier were optimized via a grid search coupled with 5-fold stratified cross-validation on the full dataset. The predefined search space included five hidden layer configurations (single layers with 50 or 100 neurons, and dual layers with 50-25, 100-50, or 100-100 neurons), L2 regularization penalty (α) values of 0.0001, 0.001, and 0.01, and initial learning rates of 0.001 and 0.01. The optimal configuration was determined to be a dual hidden layer structure with 100 and 50 neurons, α = 0.0001, and an initial learning rate of 0.001, achieving an internal cross-validation accuracy of 78.79%. These tuned parameters were subsequently utilized for the final MLP model in the outer 30-replicate 10-fold cross-validation. Finally, to ensure strict reproducibility, the random seeds for all models were globally fixed (random_state = 42 for baseline models, and random_state = 3 for the MLP to align with earlier ablation experiments).

To quantitatively assess model performance, the regression task employed the coefficient of determination ${(R}^{2})$ and the root mean square error (RMSE) as core evaluation metrics, calculated using Equations (2) and (3), respectively:

(2) $$R^{2}\;=\;1\;-\frac{\sum_{i=1}^{n}{\left(y_{i}\;-{\hat{y}}_{i}\right)}^{2}}{\sum_{i=1}^{n}{\left(y_{i}\;-\;\overset{-}{y}\right)}^{2}}$$

(3) $$\mathrm{RMSE}=\sqrt{\frac{1}{n}\sum_{i=1}^{n}{\left(y_{i}\;-\;{\hat{y}}_{i}\right)}^{2}}$$

where $y_{i}$ is the actual observed sugar content of the i-th sample, ${\hat{y}}_{i}$ is the predicted value by the model, and n is the total number of samples involved in the calculation.

Furthermore, to comprehensively evaluate the practical predictive capability of the regression models in complex field environments, the Ratio of Performance to Deviation (RPD) and the Range Error Ratio (RER) were introduced as supplementary metrics, calculated using Equations (4) and (5):

(4) $$\mathrm{RPD}=\frac{SD}{RMSE}$$

(5) $$\mathrm{RER}=\frac{Y_{max}-Y_{min}}{RMSE}$$

where SD represents the standard deviation of the reference sugar content within the sample set, and $Y_{max}$ and $Y_{min}$ denote the maximum and minimum measured sugar values, respectively.

The classification task was comprehensively evaluated using Accuracy, Area Under the Curve (AUC), and the F1-score. Accuracy represents the proportion of samples correctly graded by the model, calculated using Equation (6):

(6) $$\mathrm{Accuracy}\;=\;\frac{\mathrm{TP}\;+\;\mathrm{TN}}{\mathrm{TP}\;+\;\mathrm{TN}\;+\;\mathrm{FP}\;+\;\mathrm{FN}}$$

where TP, TN, FP, and FN denote true positives, true negatives, false positives, and false negatives, respectively.

To quantitatively validate the effectiveness of the multimodal fusion framework, this study specifically designed three progressive ablation experiment scenarios: Scenario A (fruit spectrum only), Scenario B (fruit spectrum + micro-topographic environmental features), and Scenario C (full fusion of fruit spectrum, micro-topographic environment, and plant physiological features).

## 3. Results

### 3.1. Differences in Color Indices and Spectral Features

**Figure 3:**
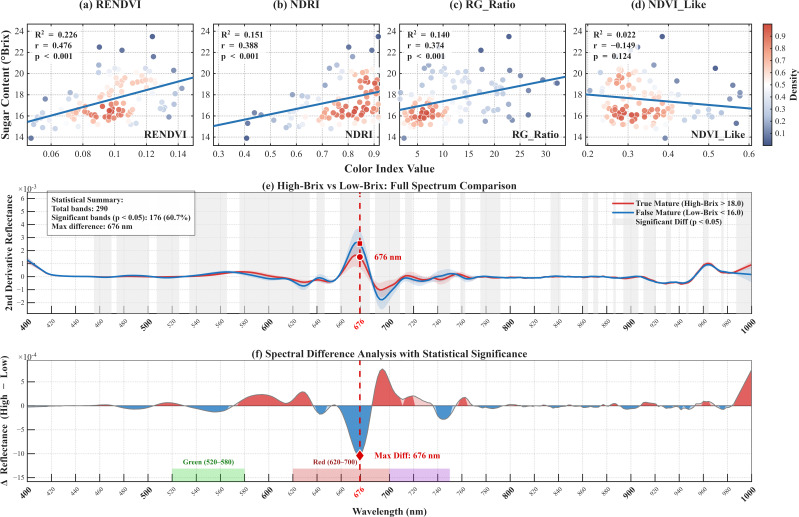
(**a**–**d**) Results of linear regression analysis for color indices (RENDVI, NDRI, RG_Ratio, and NDVI_Like); (**e**,**f**) Comparison of average spectra and spectral difference analysis between truly and falsely mature fruits, with gray areas indicating significantly different bands (*p* < 0.05).

Linear regression analysis was conducted between the color indices extracted from the hyperspectral data and the measured fruit sugar content. The results (Figure 3a–d) indicated that the Red Edge Normalized Difference Vegetation Index (RENDVI) exhibited the highest correlation, with a Pearson correlation coefficient (r) of 0.476 and a coefficient of determination ($R^{2}$) of 0.226. Further comprehensive evaluation of various common apparent color indices, such as the Normalized Difference Red Index (NDRI), Red-Green Ratio (RG_Ratio), and NDVI-like (NDVI_Like), revealed that their explained variances ($R^{2}$) were generally below 0.2. Among them, NDVI_Like exhibited the weakest correlation with sugar content and did not reach statistical significance ($R^{2}\;=\;0.022,\;r\;=\;-0.149,\;p\;=\;0.124$).

In stark contrast, the hyperspectral data themselves harbor abundant internal quality information. Using 16.0 Brix as the low-sugar threshold and 18.0 Brix as the high-sugar threshold, an independent samples t-test was performed on the full-spectrum (400–1000 nm) reflectance. The results demonstrated that across the entire spectral range, a total of 176 wavebands (accounting for 60.7%) exhibited statistically significant differences between the two sample groups (p < 0.05), as indicated by the gray shaded areas in (Figure 3e).

Specifically, in the 520–580 nm wavelength range, the reflectance of the high-sugar group was significantly lower than that of the low-sugar group; whereas in the 620–700 nm range, the exact opposite occurred, with the high-sugar group showing significantly higher reflectance. Furthermore, spectral difference analysis (Figure 3f) revealed that the maximum spectral difference between the two sample groups was precisely located at 676 nm.

### 3.2. Quantitative Regression Prediction Results for Sugar Content

To evaluate the foundational capability of hyperspectral technology in predicting sugar content, this study assessed the impact of different preprocessing methods on model performance (Table 1). The results showed that spectral data treated with the Second Derivative (2nd-Der) effectively eliminated baseline drift and light scattering effects ($R^{2}=0.222$).

**Figure 4:**
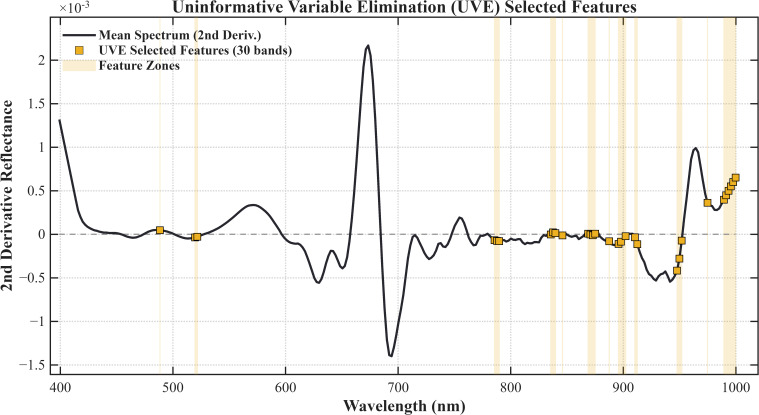
Distribution of the 30 key bands selected by UVE across the spectrum.

Building upon this, the study compared the predictive performance of the full spectrum and three feature dimensionality reduction algorithms (CARS, SPA, UVE) using a Partial Least Squares Regression (PLSR) model (Table 2). The results indicated that, compared to the 290-dimensional full-spectrum data ($R^{2}=0.222$), all three algorithms improved prediction accuracy while reducing dimensionality. The CARS algorithm extracted 90 bands ($R^{2}=0.239$); SPA extracted 4 bands ($R^{2}=0.252$) and UVE extracted 30 key bands, achieving the optimal predictive performance ($R^{2}\;=\;0.310$, RMSE = 1.574 °Brix). Consequently, this study selected the 30 characteristic wavelengths extracted by the UVE algorithm (Figure 4) as the feature inputs for subsequent modeling.

Based on the 30 extracted characteristic wavelengths, nine regression algorithms were evaluated (Table 3). The results revealed that the PLSR model yielded the highest prediction accuracy, with a cross-validated $R^{2}$ of 0.269 and an RMSE of 1.621 °Brix. The $R^{2}$ values for Random Forest and Lasso were 0.220 and 0.241, respectively. Overall, the $R^{2}$ values for all regression models did not exceed 0.30. As evaluated in Table 3, the RPD and RER values for the optimal PLSR model were restricted to 1.17 and 5.92, respectively, calculated based on the actual sample standard deviation (1.904 °Brix) and range (13.90–23.50 °Brix). In open-field coffee phenotyping, such modest regression indicators are frequently observed due to overlapping physiological absorption features and severe canopy optical interference, indicating that precise continuous numerical prediction is highly unstable under wild mountainous environments.

### 3.3. Multimodal Classification Models and Ablation Experiments

Given that the regression RPD values (maximum 1.17) indicated a structural limitation in predicting precise absolute sugar digits under severe field noise, this study shifted the problem formulation from continuous regression to threshold-based quality classification. This shift aimed to evaluate whether a binary decision logic could provide higher fault tolerance for post-harvest grading tasks.

Addressing the limitations of single-modality prediction accuracy, this study introduced a multimodal fusion framework encompassing fruit spectra, micro-topography, and plant physiology for quality classification. To determine the core classifier, a systematic evaluation of nine mainstream machine learning algorithms was conducted using 10-fold stratified cross-validation based on the full-modality feature set (36-dimensional features) (Table 4).

The results indicated that the Multilayer Perceptron (MLP) achieved a classification accuracy of 76.85%, an Area Under the Curve (AUC) of 0.825, and an F1-score of 0.667. The accuracies for Random Forest and Logistic Regression were 75.93% and 74.07%, respectively. Consequently, this study ultimately selected the MLP as the core foundational model for subsequent mechanism validation.

Multimodal feature ablation experiments were systematically performed across 30 independent replicates of 10-fold cross-validation to guarantee statistical stability (Table 5). When only the 30-dimensional fruit hyperspectral features were input (Scenario A), the model achieved a baseline mean accuracy of 75.93% (mean AUC = 0.832). Under Scenario B, where only micro-topographic features were added, the mean accuracy fluctuated to 75.56% (mean AUC = 0.818). However, when plant physiological attributes were incorporated to complete the multimodal fusion framework (Scenario C), the classification performance reached its optimum, delivering a mean accuracy of 77.22% (mean AUC = 0.827). A paired t-test confirmed that this Accuracy improvement is statistically significant over the spectral-only baseline (*p*-value = 0.017, p < 0.05), demonstrating that multimodal integration effectively enhances the model’s discriminative capability.

**Figure 5:**
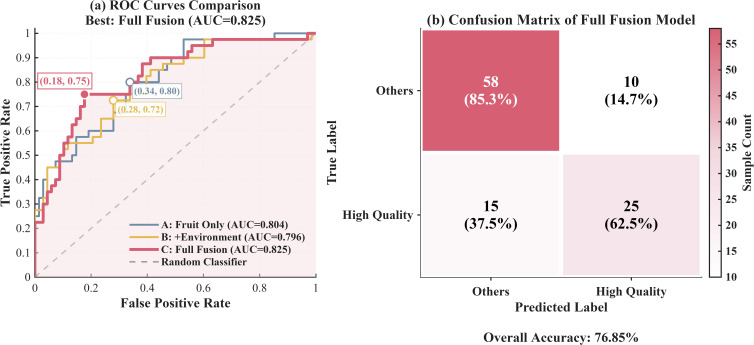
(**a**) Comparison of ROC curves for the three scenarios; (**b**) Confusion matrix of the full fusion model. Note: This figure visualizes the prediction distribution of a single representative 10-fold cross-validation run (Accuracy = 76.85%, AUC = 0.825), whereas Table 5 presents the robust statistical mean evaluated across 30 independent replicates.

The ROC curves (Figure 5a) and the confusion matrix (Figure 5b) of the full fusion model collectively demonstrate that the synergistic effect of multimodal information significantly enhances the model’s discriminative capability in complex environments.

### 3.4. Analysis of Key Factors Influencing Quality Differentiation

**Figure 6:**
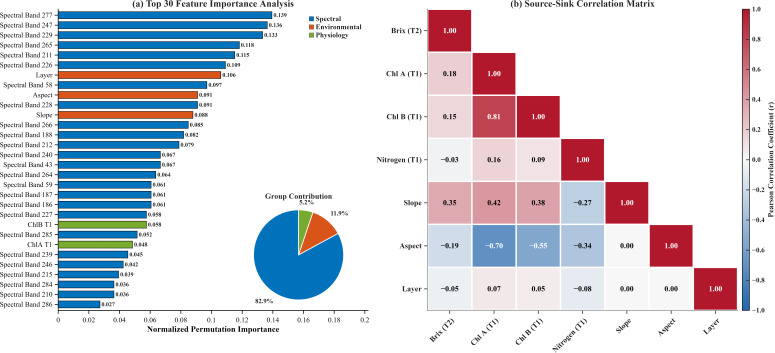

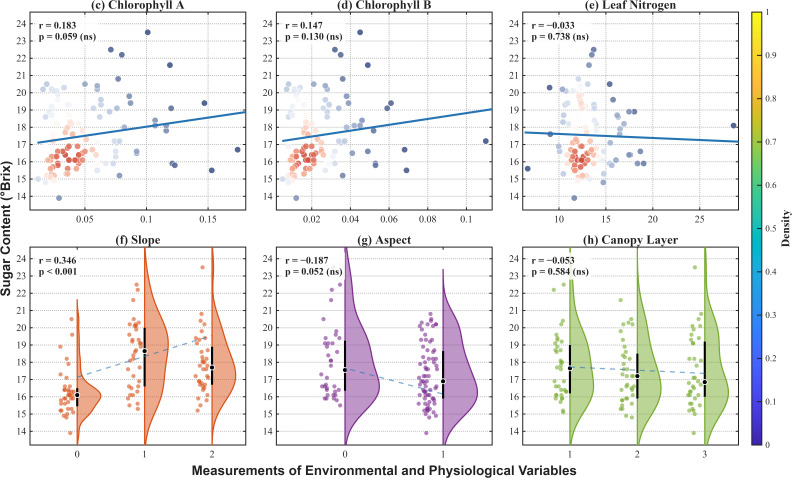
(**a**) Top 30 feature importance ranking and group contribution pie chart; (**b**) Source-Sink correlation matrix; (**c**–**e**) Scatter plots of the relationship between leaf physiological factors (Chlorophyll A, Chlorophyll B, Leaf Nitrogen) and fruit sugar content; (**f**–**h**) Scatter and violin plots of the relationship between micro-terrain factors (Slope, Aspect, Canopy Layer) and fruit sugar content. The solid lines in (**c**–**e**) and dashed lines in (**f**–**h**) represent the linear regression trends.

Feature importance analysis of the full fusion model (Figure 6a) revealed the key variables influencing the classification decisions. In addition to the dominant core spectral wavebands (accounting for 82.9% of the contribution), micro-topographic factors (such as canopy layer, aspect, and slope) exhibited prominent importance weights (accounting for 11.9%); meanwhile, plant physiological features collectively (accounting for 5.2%) also provided effective support for the model’s discriminative ability.

Furthermore, global Pearson correlation heatmap analysis demonstrated that among various non-spectral environmental and physiological factors, the micro-topographic factor ‘slope’ exhibited the highest positive correlation with fruit sugar content (Figure 6b).

As detailed in the representative scatter and violin plots (Figure 6c–h), slope showed a highly significant positive correlation with sugar content (“r = 0.346, *p* < 0.001”), while aspect exhibited a weak negative correlation trend at the margin of significance (“r = −0.187, *p* = 0.052”), and canopy layer showed no significant correlation (“r = −0.053, *p* = 0.584”). Regarding leaf physiological indices, chlorophyll A content, which reflects the plant’s photosynthetic capacity, exhibited a positive correlation trend (“r = 0.183, *p* = 0.059”), chlorophyll B also showed a weak positive correlation trend (“r = 0.147, *p* = 0.130”), while leaf nitrogen content had no significant correlation with sugar content (“r = −0.033, *p* = 0.738”).

## 4. Discussion

### 4.1. Mechanism of Color-Quality Asynchrony in Mountain Coffee

This study utilized hyperspectral imaging technology to quantitatively confirm the existence of a significant asynchronous phenomenon between the external color appearance and internal sugar accumulation of mountain coffee cherries. This finding contrasts with conclusions drawn under controlled laboratory environments, where exocarp color changes synchronously with sugar content [4].

Combining the previous full-spectrum significance analysis results, the external red wavebands (approximately 620–680 nm) relied upon for traditional harvesting precisely coincide with the regions of most significant spectral reflectance difference. As verified in color-turning studies of other fruits such as apples, the sharp changes in reflectance within this waveband (620–680 nm) are primarily attributed to the degradation of background chlorophyll [19,33]. This evidence at the physical optics level directly explains the fundamental cause of the failure in traditional visual assessment.

It can be inferred that this discrepancy stems from the inherent complex environmental conditions of mountainous systems [34]. Existing research has confirmed that slope, by regulating the incident angle of solar radiation, can generate massive differentiation in water and thermal resources at a micro-scale [35], and vertical canopy gradients further exacerbate this variation [36]. Such abiotic stresses induced by micro-topography exert differentiated regulation on the synthesis of secondary metabolic pigments and the accumulation of primary metabolic sugars in the fruit.

The synthesis of anthocyanins (which determine the red color) is extremely sensitive to light intensity [37], whereas sucrose accumulation relies more heavily on moisture conditions and the translocation of photosynthates [38]. Therefore, in environments such as steep, sun-facing slopes, the phenomenon of “false maturity”—where the epidermis rapidly turns red while internal sugar accumulation is restricted by stress—is highly prone to occur. Our data provide a robust ecophysiological explanatory framework for this production conundrum.

It is precisely this weak statistical correlation that inversely demonstrates the unreliability of traditional harvesting logic based on a single indicator (such as color) [39], thereby highlighting the irreplaceability of the deep multimodal fusion framework proposed in this paper when dealing with complex habitats [40]. Moreover, from a post-harvest perspective, the failure to intercept these falsely mature fruits during the sorting stage will directly lead to a deficiency of critical flavor precursors. Since reducing sugars are essential substrates for the Maillard reaction during the roasting process [41], the environmentally driven “false maturity” intrinsically explains the high variability in the sensory quality of mountain coffee. This highlights the irreplaceable value of the proposed deep multimodal fusion framework in ensuring raw material consistency before downstream processing.

### 4.2. Multimodal Fusion and Model Performance Optimization

The results of this study indicate that although hyperspectral imaging can detect the false maturity phenomenon, the accuracy of quantitative sugar content prediction based solely on it is limited. This predictive baseline profoundly reveals the obvious intrinsic limitations of relying exclusively on single-modality fruit signals for quantitative prediction under complex environmental stresses. This reflects the inadequacies in mechanistic interpretation and extrapolation capabilities of prediction models based purely on statistical correlation within complex agricultural systems.

The core of the multimodal fusion framework (integrating plant physiology, micro-topography, and fruit spectra) proposed in this study lies in compensating for the information deficit of single spectral signals by introducing environmental and physiological background information. Traditional spectral modeling typically assumes that the mapping relationship between spectra and sugar content is globally constant [42,43]. However, in the field, illumination conditions, canopy architecture, and the complex three-dimensional geometric structure of the plants all exert nonlinear interferences on the spectral reflectance of the fruits [44].

As observed from the ablation experiment results, when only micro-topographic features were introduced without the constraints of plant physiological status, the complex terrain factors paradoxically interfered with the model’s discrimination as background noise, reflecting the complex nonlinear relationship between environmental factors and spectral signals.

Incorporating terrain factors and plant physiological features essentially provides the model with prior knowledge correction. For the future development of portable post-harvest sorting equipment, if the ‘micro-topographic origin’ (e.g., steep slope vs. gentle slope) and ‘leaf nutritional status’ of the batch materials can be input as prior calibration parameters, it will effectively decouple the spectral noise brought by the complex environment. This strategy significantly improves the generalization ability of the sugar detection model across different harvested batches. Furthermore, this framework effectively explains and addresses the phenomenon of low single-variable correlation observed in our data. As highlighted by the correlation matrix, there is an absence of high linear correlation between sugar content and the individual spectral, source, or environmental variables. This low linear correlation is consistent with the highly complex, nonlinear nature of mountainous ecosystems, although other factors such as limited sample size and measurement variability may also contribute. In such heterogeneous habitats, micro-topographic stresses and physiological variations do not drive sugar accumulation in a simple independent manner; instead, they interact dynamically, creating severe overlapping optical noise that obscures direct linear mapping. Because these underlying biological interactions are highly nonlinear, traditional continuous regression models struggle to isolate the target signal (as evidenced by the restricted regression RPD of 1.17). To benchmark this value against the literature, Jin et al. [45] recently stated that an RPD greater than 2.5 is indicative of accurate prediction in hyperspectral applications, and Wang et al. [20]. achieved an RPD of 2.57 for SSC prediction in apples under controlled laboratory conditions. The marked difference between 2.57 (apple, controlled) and 1.17 (coffee, mountainous field) is not due to instrumentation or algorithms, but rather reflects the severe environmental noise inherent to mountain coffee systems—overlapping canopy reflectance, micro-topographic stresses, and variable illumination. This comparison further underscores why precise continuous regression is structurally unattainable under such conditions. Consequently, this explicitly necessitates the adoption of deep nonlinear algorithms (like the MLP) and justifies our strategic shift toward a threshold-based multimodal classification framework. From an industrial perspective, sorting machinery operates on a binary decision logic rather than precise numerical prediction. By capitalizing on boundary decision margins to absorb field noise, the full fusion classification model successfully unlocked the predictive synergy of the “Source-Environment-Sink” loop, significantly improving the classification accuracy to 77.22% (p = 0.017) and effectively optimizing the operational decision boundary. Compared to previous studies that solely utilized coffee bean spectra for origin and variety classification [13], this study targeted the in situ field assessment of fresh cherries, which exhibits significantly greater variability. The results demonstrate that when spectral signals become obscured by environmental noise, the leaf nutritional status (Source) and micro-topographic conditions (Environment) can assist the model in delineating the boundaries of high-quality fruits more clearly within the feature space.

Particularly, the Multilayer Perceptron network comprehensively outperformed traditional classifiers such as Support Vector Machines and Random Forests in the lateral baseline testing of the full fusion scenario, further substantiating the significant advantage of this network architecture in capturing the deep nonlinear mapping relationships of cross-modal heterogeneous features. This coincidentally aligns with the “context-aware” concept emphasized in the recent computer vision field [46,47], and concurrently represents the core direction advocated by current agricultural phenomics to address the complexities of genotype-environment-management interactions [48].

### 4.3. Topographic Drivers of Fruit Quality Variation

The analyses in this study consistently point to ‘slope’ as a core environmental driving factor. The positive driving effect of slope on sugar content reflects the redistribution effect of topography on water and thermal resources [49]. Furthermore, continuous slope gradients significantly impact plant physiological processes and resource allocation by regulating soil moisture and microclimate conditions [34,50]. For instance, steeper slopes are typically accompanied by reduced soil moisture and nitrogen content, whereas gentle slopes are more conducive to the accumulation of water and nutrients [51].

This finding bears direct agronomic management implications: the quality variation in mountainous coffee orchards is not randomly distributed but is systematically driven by micro-topography. Integrating the results from the global feature importance analysis and variable correlations, this study further refines the logical closed-loop of quality regulation under complex mountainous habitats: specifically, slope is associated with the redistribution of micro-scale resources such as light, heat, and water, which may contribute to differentiated responses in the photosynthetic capacity of source leaves [52]; this alteration in the plant’s nutrient supply capacity ultimately cascades and amplifies the spatial differentiation of primary metabolites (i.e., sugars) within the fruit [53].

This cross-scale cascading regulatory effect is precisely the fundamental ecological mechanism that causes harvest decisions relying solely on external appearance and color to fail. This provides a scientific basis for implementing differentiated site-specific management, marking a crucial step towards realizing precision agriculture [54].

### 4.4. Research Limitations

Although this study has validated the effectiveness of the multimodal fusion framework, several limitations remain.

First, the external validation of the model’s generalization capability is currently insufficient. Constrained by the difficulty of sample acquisition, the conclusions of this study are presently based on data from a single growing season, a single geographical region (Lujiangba, Baoshan), a specific clone of Typica (a single vegetatively propagated clonal lineage), without the introduction of an independent external validation set across different producing regions, other Typica clones or varieties. Moreover, the studied Typica clone exhibits high susceptibility to pests and diseases under field conditions, which may influence leaf physiological traits (e.g., chlorophyll content) and potentially alter fruit spectral signatures. Therefore, the proposed multimodal model is specifically validated on this stress-sensitive clone in a real mountainous environment; its transferability to more robust or disease-resistant Typica clones, or to other Arabica varieties, requires further investigation. Future research must further test the spatiotemporal transferability of the multimodal framework through multi-site and multi-variety trials.

Second, the dimensionality of environmental features requires deepening. The micro-topographic features selected in this study were primarily static terrain factors such as slope and aspect; while capable of reflecting the redistribution of water and heat resources, they fail to capture real-time dynamic microclimate data (e.g., real-time temperature differences, rainfall intensity) during the critical developmental windows that affect sugar accumulation. The lack of coupling with dynamic meteorological factors may limit the model’s ability to capture the interaction mechanisms between quality and environment under extreme weather events.

Third, regarding tissue-specific characterization, the reference sugar content (Brix) in this study was derived from the pulp and mucilage, excluding the bean endosperm. While this is the standard field phenotyping method and accurately reflects the sugar reservoir available for post-harvest fermentation, it inherently acts as an indirect proxy for the biochemical composition of the final green bean. Future research should integrate laboratory-based destructive chemical assays (e.g., High-Performance Liquid Chromatography, HPLC) to directly quantify sucrose accumulation within the bean endosperm, thereby further elucidating the internal physiological mechanism of “false maturity.”

Moreover, this study preliminarily explored the feasibility of using multimodal data (fruit spectra and plant physiological characteristics) from the color turning initiation stage (T1) to achieve early prediction of fruit quality at the fully mature stage (T2). However, experimental results indicated that early prediction models based on current static features performed poorly (the coefficients of determination for all regression models were less than 0, and the AUCs of classification models were close to the 0.5 random baseline). This result provides a crucial agronomic insight: the complex mountainous micro-topographic environment exerts a nonlinear, cumulative effect on the physiological evolution of fruits from the color turning stage to the harvesting stage. Relying solely on static multimodal features extracted at specific growth nodes fails to effectively capture the complete dynamic evolution process of sugar accumulation.

Future studies could attempt to integrate multi-sensor platforms borne by Unmanned Aerial Vehicles to achieve synchronous, high-frequency monitoring of phenotypic and environmental data at the orchard scale. Furthermore, integrating mechanistic models (such as crop growth models) with deep learning algorithms could facilitate an evolution from pure data-driven approaches to dual-driven (mechanism and data) methodologies, aiming to thoroughly decipher the biological essence of the false maturity phenomenon in mountainous crops.

Additionally, due to randomized sample-level splitting in cross-validation, observations from different canopy layers of the same tree could potentially be distributed across both training and validation folds simultaneously, posing an overfitting risk. Future trials should employ spatial GroupKFold strategies. Furthermore, executing feature selection algorithms (UVE) on the global dataset poses a minor data leakage risk. However, this global approach was deliberately chosen for ‘biomarker discovery’ to ensure the steady extraction of physical wavebands representing the Typica cultivar, as fold-specific subsets would fluctuate and lose physical interpretability.

## 5. Conclusions

Targeting the asynchronous phenomenon between external color turning and internal sugar accumulation in mountain coffee cherries, this study constructed a multimodal quality discrimination framework integrating fruit hyperspectral imaging, micro-topography, and plant physiological characteristics. The primary conclusions are as follows:

(1) Quantitatively confirming the risk of misclassification in industrial sorting due to “false maturity.” The spectral differences between high- and low-sugar fruits are highly concentrated in the red and red-edge regions (maximized at 676 nm), which confirms from a physical optics perspective the unreliability of harvesting decisions relying solely on external color in complex habitats.
(2) Multimodal fusion significantly enhances discrimination accuracy. Compared to the single-spectrum model (mean accuracy of 75.93%), the fully fused MLP model incorporating topographic and physiological features effectively demonstrates the potential to mitigate environmental noise interference, improving the mean classification accuracy to 77.22% with a mean AUC of 0.827.
(3) Establishing a topography-aware calibration strategy for coffee quality assessment. Correlation analysis confirms that micro-topographic slope (r = 0.346, p < 0.001) is the key driving factor for the spatial differentiation of fruit sugar content, while plant chlorophyll A content (r = 0.183, p = 0.059) exhibits a corresponding physiological response trend. This study provides preliminary theoretical and data support for the intelligent sorting of raw materials and demonstrates the potential to ensure the post-harvest flavor consistency of mountainous crops.

However, these findings are based on a single growing season, one geographical location (Lujiangba, Baoshan), and a single Typica clone. The generalizability of the proposed multimodal framework to other regions, seasons, or Arabica varieties (including other Typica clones) remains to be validated using independent external datasets. Future multi-site and multi-clone trials are therefore necessary before practical deployment in industrial sorting lines.

## 6. Patents

The work reported in this manuscript has resulted in a patent application in China.

Patent Title: A Non-destructive Assessment Method and System for Sugar Content of Mountain Coffee Fruits Based on Multimodal Fusion.

Patent Application Number: CN 202610396314.0.

Applicant: Yunnan Agricultural University.

Inventors: Hongbo Zhao, Zhijia Wang, Zhiyong Cao, Linrui Deng, Huijuan Yang, Luoyi Zheng, and Changjun Deng.

## Figures and Tables

**Table 1:** Performance comparison of different spectral preprocessing methods for Brix prediction using PLSR.

Preprocessing Method	Optimal Components	R^2^	RMSE	RPD	RER
2nd-Der	3	0.222	1.671	1.14	5.75
1st-Der	1	0.215	1.680	1.13	5.71
Detrend	5	0.200	1.695	1.12	5.66
Normalize	4	0.184	1.712	1.11	5.61
SNV	7	0.174	1.722	1.11	5.57
MSC	2	0.173	1.724	1.10	5.57
Raw	3	0.163	1.733	1.10	5.54
MA	3	0.163	1.733	1.10	5.54
SG-Smooth	3	0.163	1.734	1.10	5.54

**Table 2:** Performance comparison of different feature selection algorithms.

Method	Selected Bands	R^2^	RMSE	RPD	RER
UVE	30	0.310	1.574	1.21	6.10
SPA	4	0.252	1.639	1.16	5.86
CARS	90	0.239	1.654	1.15	5.80
Full-Spectrum	290	0.222	1.671	1.14	5.75

**Table 3:** Performance comparison of different regression models on UVE-selected feature subset.

Model	R^2^	RMSE	RPD	RER
PLSR	0.269	1.621	1.17	5.92
Lasso	0.241	1.651	1.15	5.81
Ridge	0.234	1.659	1.15	5.79
Random Forest	0.220	1.674	1.14	5.73

**Table 4:** Performance comparison of different machine learning classifiers based on the full-fusion multimodal framework.

Model	Accuracy (%)	AUC	F1-Score
MLP (proposed)	76.85	0.8250	0.6667
Random Forest	75.93	0.8072	0.6389
Logistic Regression	74.07	0.8015	0.6818
Gradient Boosting	73.15	0.7665	0.6329
XGBoost	72.22	0.7912	0.6429
LightGBM	71.30	0.7835	0.6265
SVM (RBF)	68.52	0.7169	0.6304
Decision Tree	65.74	0.6456	0.5647
K-NN (k = 5)	63.89	0.6908	0.4935

**Table 5:** Performance comparison of multi-modal ablation experiments using MLP classifier.

Scenario	Description	Mean AUC	Mean Accuracy (%)	Paired *t*-Test *p*-Value	Significance
A	Fruit spectrum only	0.832	75.93	-	Reference
B	Spectrum + Environment	0.818	75.56	0.424	Not Sig
C	Full fusion (Spectrum + Env + Leaf)	0.827	77.22	0.017	* (Significant)

* indicates statistical significance at the p < 0.05 level compared to the baseline Scenario A. The *p*-value was calculated specifically for Accuracy.

## Data Availability

The original contributions presented in this study are included in the article. Further inquiries can be directed to the corresponding author.

## Abbreviations

The following abbreviations are used in this manuscript:

HSI	Hyperspectral Imaging
MLP	Multilayer Perceptron
UVE	Uninformative Variable Elimination
PLSR	Partial Least Squares Regression
SVM	Support Vector Machine
RF	Random Forest
AUC	Area Under the Curve
ROC	Receiver Operating Characteristic
